# Identification of gingipain-specific I-A^b^-restricted CD4^+^ T cells following mucosal colonization with *Porphyromonas gingivalis* in C57BL/6 mice

**DOI:** 10.1111/omi.12038

**Published:** 2013-08-14

**Authors:** PD Bittner-Eddy, LA Fischer, M Costalonga

**Affiliations:** Division of Periodontology, Department of Developmental and Surgical Sciences, School of Dentistry, University of MinnesotaMinneapolis, MN, USA

**Keywords:** animal model, major histocompatibility complex class II tetramer, oral mucosal colonization, periodontitis, *Porphyromonas gingivalis*, virulence

## Abstract

Chronic periodontitis is associated with *Porphyromonas gingivalis* infection. Although virulence factors of *P. gingivalis* are hypothesized to contribute to the pathogenesis of periodontitis, it is unclear whether the local CD4^+^ T-cell-mediated response they elicit prevents or contributes to periodontal bone destruction. We hypothesize that major histocompatibility complex class II I-A^b^-binding peptides existing in Kgp and RgpA are presented to CD4^+^ T cells during *P. gingivalis* oral colonization. The protein sequences of gingipains RgpA and Kgp, and OMP40 and OMP41 of *P. gingivalis* were scanned using an I-A^b^-binding matrix. From this analysis we identified 53 candidate peptides that had the potential to engage the peptide-binding groove of the I-A^b^ molecule of C57BL/6 mice. An ELISpot-based screen revealed those peptide-primed effector/memory CD4^+^ T cells that could be re-stimulated *in vitro* with *P. gingivalis* or the peptide itself to produce interleukin-17A or interferon-γ. Two immunodominant peptides, Kgp_467–477_ (pKgp) and RgpA_1054–1064_/Kgp_1074–1084_ (pR/Kgp) were identified and engineered to be displayed on I-A^b^ molecular tetramers. Peptide pR/Kgp is conserved across all sequenced *P. gingivalis* strains. C57BL/6 mice were orally inoculated with *P. gingivalis* strain 53977 and cervical lymph node cells were stained with phycoerythrin-conjugated pKgp::I-A^b^ and pR/Kgp::I-A^b^ tetramers. We found that only pR/Kgp::I-A^b^ bound with the desired specificity to gingipain-specific CD4^+^ T cells. The pR/Kgp::I-A^b^ tetramer complex will allow the identification of effector/memory CD4^+^ T cells specific for two virulence factors of *P. gingivalis* strains associated with periodontal disease.

## Introduction

Chronic periodontitis is linked to the presence of a subgingival microbial biofilm that stimulates persistent inflammation of the marginal gingiva and is characterized by the destruction of the periodontium, resorption of alveolar bone and subsequent loss of teeth (Oliver & Brown, 1993; Oliver *et al*., 1998). This subgingival biofilm is a consortium of microbial species that includes the gram-negative, anaerobic bacterium *Porphyromonas gingivalis* (Socransky *et al*., 1998; Tran & Rudney, 1999; Lamont & Jenkinson, 2000). Severe periodontitis and periodontal disease progression have been associated with *P. gingivalis* colonization of the oral mucosa and gingival sulcus (Yang *et al*., 2004; Byrne *et al*., 2009). Therapeutic interventions that reduce the burden of *P. gingivalis* in patients suffering from periodontitis result in restoration of gingival health, but without a concomitant regeneration of the destroyed periodontium (Van Dyke *et al*., 1988).

Bacterial colonization of the gingival sulcus and organization of the bacterial plaque biofilm induce an initial robust innate immune response that primarily involves recruitment and transepithelial migration of neutrophils and a strong inflammatory response characterized by secretion of pro-inflammatory cytokines such as interleukin-6 (IL-6), tumor necrosis factor-α and interferon-γ (IFN-γ) (Dixon *et al*., 2004). Adaptive immune responses through activated CD4^+^ T helper cells and secretion of immunoglobulin by B cells also play a part in containment of the biofilm but, paradoxically, may also be drivers of disease progression (Baker *et al*., 1999, 2002). In a murine model of periodontitis, adaptive immune responses, primarily through effector CD4^+^ T helper cells, were found to contribute extensively to *P. gingivalis*-induced alveolar bone loss (Baker *et al*., 1999, 2002). The role that different CD4^+^ T helper cell subsets play in the pathogenesis of periodontal diseases remains unresolved (Gemmell *et al*., 2007).

Significant levels of *P. gingivalis*-specific immunoglobulins have been found in the sera of patients with ongoing periodontal disease (Ebersole *et al*., 1987). The antibody response and effector function of the immunoglobulin molecule are preceded by CD4^+^ T helper cell differentiation. Therefore, the pro-inflammatory effector functions of isotype-switched immunoglobulins via complement activation and opsonization of the oral biofilm are essentially a result of the CD4^+^ T helper cell phenotype. In patients with active periodontitis, it is unclear if effector CD4^+^ T helper cells ensure disease protection via macrophage activation/neutrophil recruitment and antibody-mediated immunity or whether such a heightened immune response drives osteoclastogenesis and, consequently, periodontal destruction (Gemmell *et al*., 2007).

Despite a number of reports that describe the identification of B-cell epitopes within important *P. gingivalis* virulence factors (O'Brien-Simpson *et al*., 2005; Frazer *et al*., 2006; Miyachi *et al*., 2007; Sharma *et al*., 2007), the nature of the specific protective CD4^+^ T-cell response against such proteins remains unknown in humans and is poorly understood in mice (Tam *et al*., 2008). Bridging this gap in our knowledge is critical to our understanding of the pathogenesis of periodontitis and to the development of an effective protein-based vaccine against *P. gingivalis*. To be effective, such a vaccine must be capable of activating CD4^+^ T cells that can in turn stimulate the B cells that produce a protective antibody response against *P. gingivalis*. Identification of the relevant CD4^+^ T-cell epitopes, within vaccine candidates, in humans is problematic because humans have multiple allelic forms of major histocompatibility complex (MHC) class II molecules capable of displaying microbial peptides. Inbred mouse strains, however, have a limited repertoire of MHC class II alleles and make an excellent tool for screening potential CD4^+^ T-cell epitopes.

In this current work, we choose C57BL/6 mice to screen *P. gingivalis* proteins for immunodominant CD4^+^ T-cell epitopes. The C57BL/6 strain is well characterized as a murine model of periodontitis (Baker *et al*., 2000a) and has an advantage over the BALB/c strain, for example, in that it only displays a single MHC class II molecule (I-A^b^). By restricting the MHC class II molecule in this way we reduced the number of potential microbial CD4^+^ T-cell epitopes that can be displayed and that are able to be recognized by the repertoire of CD4^+^ T-cell receptors found in the C57BL/6 strain. Moreover, proven methodologies exist for generating pMHCII tetramers based on the I-A^b^ molecule rather that I-A^d^ or I-E^d^. This capability will allow us to construct pMHCII tetramers incorporating immunodominant CD4^+^ T-cell epitopes identified herein and use these tetramers to track and phenotype antigen-specific CD4^+^ T cells in mice expressing the I-A^b^ molecule (Moon *et al*., 2007, 2009).

We targeted *P. gingivalis* gingipains and two highly expressed outer membrane proteins of the OmpA superfamily that are highly conserved and that are potentially immunogenic in mice (Ross *et al*., 2004). Gingipains are arginine- or lysine-specific cysteine proteinases that are implicated in the proteolytic breakdown of immunoglobulins, matrix proteins and surface receptors found on cells resident in the gingiva, the degradation and inactivation of inflammatory cytokines, and have been associated with disease progression and periodontal bone destruction in animal models of periodontitis (O'Brien-Simpson *et al*., 2003). We hypothesize that I-A^b^-binding peptides existing in Kgp and RgpA are presented to CD4^+^ T cells during *P. gingivalis* oral colonization. Furthermore, we predict that such epitopes will allow us to construct pMHCII tetramers that can be used as a tool to track and phenotype specific CD4^+^ T cells activated after oral infection with *P. gingivalis*.

## Methods

### Mice

C57BL/6J mice (H-2^b^) were purchased from The Jackson Laboratory (Bar Harbor, ME) and housed in microisolator cages in accordance with University of Minnesota and National Institutes of Health guidelines. All experiments were performed on age-matched (6–8 weeks) and sex-matched mice using protocols approved by the Institutional Animal Care and Use Committee of the University of Minnesota.

### *In silico* identification of potential immunodominant peptides

The amino acid (aa) sequence of cysteine proteinase gingipains RgpA (PGN_1970, 1703 aa) and Kgp (PGN_1728, 1723 aa), and putative outer membrane proteins OMP40 (PGN_0728, 380 aa) and OMP41 (PGN_0729, 391 aa), were retrieved from the NCBI database containing the complete annotated sequence of *P. gingivalis* strain ATCC 33277 (Naito *et al*., 2008). Each protein sequence was scanned using the Wilson–Rudensky (W-R) scoring matrix (Zhu *et al*., 2003) to identify 9-mer peptides with the potential to fit the peptide-binding groove of the murine MHC class II I-A^b^ molecule. Peptides that had an aggregate score of 50 or more were synthesized (GenScript, Piscataway, NJ) and their ability to invoke CD4^+^ T-cell responses in ELISpot experiments was determined.

### Mice inoculations

Mice were injected subcutaneously at three points along each flank with a total of 3 × 10^9^ colony-forming units (CFU) of live *P. gingivalis* strains ATCC 53977, W50 or DPG3 prepared in de-gassed phosphate-buffered saline (PBS), sham inoculated with a similar volume of PBS, or injected with 25 μg lipopolysaccharide in incomplete Freund's adjuvant with or without (vehicle control) pooled peptide. These strains were chosen for their ability to induce alveolar bone loss in a murine model of periodontitis (Baker *et al*., 2000a). Peptide pools resuspended in dimethyl sulfoxide and emulsified, contained 100 μg of each constituent peptide. Mice were sacrificed 14 days after inoculation and lymph nodes draining the subcutaneous injection sites and spleens were harvested for ELISpot. In subcutaneous immunization experiments, mice were re-challenged with live *P. gingivalis* 10 days after the primary inoculation so as to boost CD4^+^ T-cell responses. In experiments requiring oral infection, mice were pre-treated with antibiotics and fed 4 × 10^9^ CFU *P. gingivalis* in 2% carboxymethylcellulose or vehicle control by oral gavage, six times, 4 days apart as previously described (Baker *et al*., 1999). Blood was drawn on day 12 to check for immunoglobulins specific against *P. gingivalis* using ELISA and paper-point samples were taken from the oral cavity and plated on sheep blood agar plates to confirm *P. gingivalis* colonization. Mice were sacrificed 14 days after their last oral feed and draining cervical lymph nodes were harvested for detection of antigen-specific CD4^+^ T cells.

### ELISpot

Single-cell preparations from lymph nodes and spleens of control or inoculated mice were prepared and CD4^+^ T cells were purified by negative selection using magnetic cell sorting according to the manufacturer's recommended protocol (Miltenyi Biotec, Auburn, CA). We routinely found CD4^+^ T cells as > 94% of the purified cell populations when test samples were analysed by flow cytometry following cell-surface staining with anti-mouse-CD3ε-fluorescein isothiocyanate (145-2C11; eBioscience, San Diego, CA) and anti-mouse-CD4-Peridinin chlorophyll protein (RM4-5; BD Biosciences Pharmingen, San Diego, CA) antibodies. Naive mice were used as a source of splenocytes for co-cultivation with purified CD4^+^ T cells. Single-cell suspensions of splenocytes were irradiated using an X-Rad 320 Biological Irradiator (Precision X-Ray, North Branford, CT) delivering sufficient irradiation (2000 rads) to inhibit cell proliferation and cytokine expression capabilities while maintaining MHC class II antigen presentation function. ELISpot assays were performed using Millipore Multiscreen 96-well filtration plates (EMD Millipore, Billerica, MA). Plates were pre-coated with cytokine capture antibodies specific for mouse IFN-γ or IL-17A (eBioscience). Then, 5 × 10^5^ purified CD4^+^ T cells from control or inoculated mice were combined with 3 × 10^5^ γ-irradiated naive splenocytes, as a source of antigen-presenting cells (APCs), in a final culture volume of 200 μl per well (Eagle's Ham's amino acids culture medium supplemented with 10% volume/volume heat-inactivated fetal calf serum, 2 mm glutamine, 0.1 mm 2β-mercaptoethanol, 100 μg ml^−1^ streptomycin, 100 IU ml^−1^ penicillin and 25 μg ml^−1^ gentamicin). For CD4^+^ T-cell stimulation, cells were incubated with either Concanavalin A (2.5 μg ml^−1^) as a positive control, un-related or no peptide as negative controls, ‘re-called’ using peptide sub-pools or a single peptide (each peptide at final concentration of 25 μg ml^−1^), or 1 × 10^8^ CFU of *P. gingivalis* strains ATCC 53977, W50 and DPG3. All microorganisms had been killed by freezing in O_2_-saturated PBS. ELISpot plates were incubated at 37°C, 5% ambient CO_2_, for 46 h in a humidified incubator then each well was washed five times with 200 μl wash buffer [1 × Dulbecco's phosphate-buffered saline (DPBS), 0.05% Tween-20] followed by two rinses with Milli-Q water. For ELISpot plate development, plates were incubated with biotinylated anti-IFN-γ or IL-17A detection antibodies (eBioscience) (50 ng in 100 μl 1 × DPBS, 10% fetal bovine serum per well) at room temperature for 3 h, washed six times with 200 μl wash buffer, twice with 200 μl 1 × DPBS then incubated with streptavidin-conjugated horseradish peroxidase (eBioscience) (100 μl of a 1/1000 dilution in 1 × DPBS, 10% fetal bovine serum) for 45 min protected from light. Wells were washed four times with 200 μl wash buffer and 2 × with 200 μl 1 × DPBS. Finally, cytokine-positive cells were revealed as discrete spots following incubation with 100 μl 3-amino-9-ethylcarbazole substrate solution (BD Biosciences, San Jose, CA) at room temperature for 20 min and enumerated using ImmunoSpot software coupled to an ELISpot plate reader (Cellular Technology Limited, Cleveland, OH). One-tailed Student's *t*-test was used to determine statistical significance between sample means.

### Production of pKgp:I-A^b^ and pR/Kgp:I-A^b^ tetramers

Sequences encoding the pKgp and pR/Kgp peptides, optimized for *Drosophila* codon usage, were cloned into modified pRMHa-3 vectors expressing a 6× His tagged version of the murine I-A^b^ β-chain gene under control of the copper sulfate-inducible metallothionein promoter (Moon *et al*., 2007). The resultant N-terminal I-A^b^ β-chain fusions were co-expressed with the α-chain of I-A^b^ in *Drosophila* S2 cells using a *Drosophila* Expression System kit (Invitrogen, Carlsbad, CA). Briefly, competent S2 cells were co-transfected with plasmids encoding either the pR/Kgp:I-A^b^ or pKgp:I-A^b^ β-chain fusion construct, a modified I-A^b^ α-chain containing a C-terminal BirA biotinylation signal, the *BirA* gene from *Escherichia coli*, and a blasticidin-resistance gene at molar ratios of 9 : 9 : 9 : 1, respectively. Selection, large-scale production of stably transformed S2 cells and induction of I-A^b^ α-chain and I-A^b^ β-chain expression was as essentially described by Moon *et al*. (2007) with the exception that the BirA substrate, D-biotin (2 μg ml^−1^), was added contemporaneously with the copper sulfate (800 μm). Resultant pR/Kgp:I-A^b^ and pKgp:I-A^b^ monomers were purified from S2 cell culture supernatants 12 days post-induction by nickel affinity chromatography using a Novagen His-Bind Purification Kit (EMD Millipore) targeting the 6× His tag engineered on the I-A^b^ β-chain, followed by size exclusion chromatography on a Sephacryl S-300 sizing column (GE Healthcare Biosciences, Piscataway, NJ). A competition titration assay was used to empirically determine what percentage of each monomer had been successfully biotinylated *in vivo*. Tetramers were generated by mixing a saturating amount of biotinylated pR/Kgp:I-A^b^ or pKgp:I-A^b^ monomer with strepavidin-conjugated phycoerythrin (ProZyme, Hayward, CA) at a molar ratio of 5–6 : 1 and incubating the mix for 1 h at room temperature. Tetramer stocks were diluted to 1 μm based on the strepavidin moiety and stored at 4°C protected from light.

### Detection of pR/Kgp or pKgp-specific CD4^+^ T cells by flow cytometry

Draining lymph nodes from inoculated mice were harvested, pooled and single cell suspensions were prepared. Then, 4 × 10^6^ lymphocytes were stained with 5–25 nm of pR/Kgp:I-A^b^ or pKgp:I-A^b^ tetramer in a final volume of 100 μl for 1 h in a room temperature (22–24°C) water bath followed by surface staining on ice for 30 min with the following panel of antibodies to unambiguously identify antigen-experienced CD4^+^ T cells: B220-eFluor450 (RA3-6B2; eBioscience), Gr-1-Pacific Blue (RB6-8C5; BioLegend, San Diego, CA), CD3ε-fluorescein isothiocyanate (145-2C11; eBioscience), CD4-Peridinin chlorophyll protein (RM4-5; BD Pharmingen), CD8α-Pacific Orange (5H10; Caltag, Carlsbad, CA) and CD44-allophycocyanin-Cy7 (IM7; BioLegend). Cells were analysed on an LSRII flow cytometer (BD Biosciences). Analysis of flow cytometry data was performed using FlowJo software (TreeStar, Ashland, OR). For enrichment of pR/Kgp-specific CD4^+^ T cells, 20 × 10^6^ lymphocytes were stained with 25 nm of pR/Kgp:I-A^b^ tetramer as described above followed by incubation with 50 μl of anti-PE-conjugated magnetic microbeads (Miltenyi Biotec) as described by Moon *et al*. (2009). The pR/Kgp:I-A^b^ tetramer stained cells were enriched using Miltenyi LS columns mounted in a MidiMACS magnet apparatus, surfaced stained, prepared for flow cytometry and analysed using FlowJo software as described above.

## Results

### Selection of suitable proteins from *P. gingivalis*

To increase the likelihood of identifying immunodominant CD4^+^ T-cell epitopes derived from *P. gingivalis*, we chose to work with *P. gingivalis* proteins that are abundantly expressed and that are anchored within the membrane and/or are secreted. We selected two well-characterized virulence factors, lysine-gingipain (Kgp 1723 aa; Accession no. BAG34247) and arginine-gingipain A (RgpA 1703 aa; Accession no. BAG34488), and two outer membrane proteins (OMP40 380 aa; Accession no. AAD51068.1 and OMP41 391 aa; Accession no. AAD51067.1) that fit these criteria. These proteins also have the advantage of being highly conserved across a number of sequenced *P. gingivalis* strains (O'Brien-Simpson *et al*., 2003; Ross *et al*., 2004), including those considered to be most virulent in a murine model of periodontitis (Baker *et al*., 2000b). Furthermore, the high degree of homology between RgpA and Kgp has the additional benefit of allowing us to identify potential CD4^+^ T-cell epitopes that are shared between these two important virulence factors.

### Relatively few peptides are predicted to fit the I-A^b^ peptide-binding groove with high affinity

We used an I-A^b^ peptide-fitting algorithm (W-R) to identify potentially immunodominant CD4^+^ T-cell epitopes (Zhu *et al*., 2003). The W-R algorithm analyses the sequence of a protein in blocks of nine amino acids using a sliding window of one and assigns an aggregate score to the 9-mer peptides based on the probability of a particular amino acid occupying the anchoring pockets of the I-A^b^ molecular groove at positions P1, P4, P6, P7 and P9. Amino acids at positions P2, P3, P5 and P8 were not scored because at these positions they would be oriented away from the I-A^b^ molecular groove and toward the T-cell receptor. For example, Kgp was scored for 1715 possible 9-mer peptides with most of them not meeting the cutoff criteria of a W-R score of at least 50 (see below). Given that most peptides eluted from I-A^b^ are longer than nine amino acids (Dongre *et al*., 2001), we chose to extend each candidate peptide by adding the two preceding amino acids to the N-terminal end. We predicted that these longer peptides would be more likely to be displayed by the I-A^b^ molecule as immunogenic peptides in our ELISpot assays. However, one consequence is that each 11-mer now has three potential fitting registers within the I-A^b^ peptide-binding groove. One register defined by the W-R score beginning at P1, and two others beginning at positions –P1 and –P2. Any 11-mer peptides that had alternative I-A^b^ binding registries with W-R score greater than 40 were not synthesized because the generation of a unique pMHCII tetramer would not be guaranteed.

**Figure 1:**
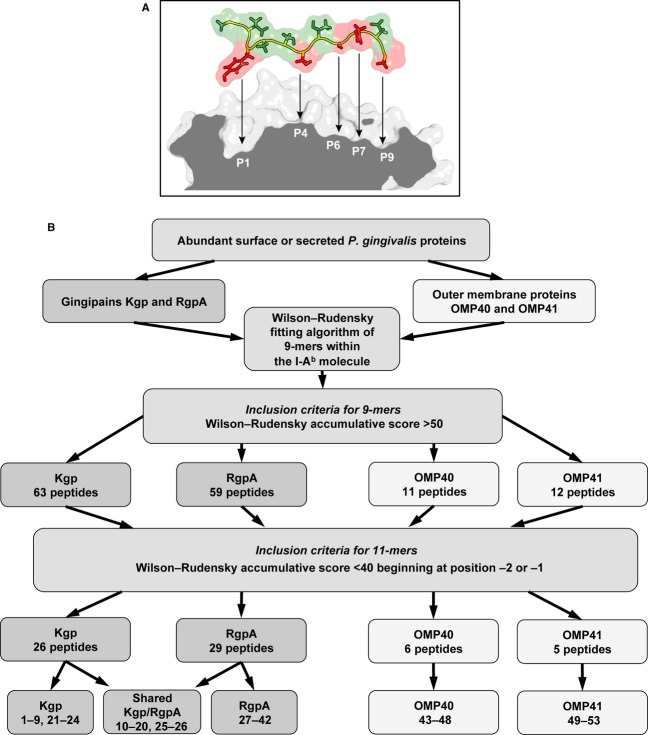
*In silico* identification of 53 potentially immunogenic CD4^+^ T-cell epitopes among *Porphyromonas gingivalis* proteins Kgp, RgpA, OMP40 and OMP41. (A) Three-dimensional representation of peptide pR/Kgp engaging the peptide-binding groove of the murine major histocompatibility complex class II I-A^b^ molecule. Amino acids at positions 1, 4, 6, 7 and 9 (colored red) engage directly with pockets formed in I-z^b^ and so determine the avidity and specificity of the interaction with I-A^b^. Amino acids at positions -1, -2, 2, 3, 5 and 8 (colored green) are oriented outwards and so contribute to CD4^+^ T-cell specificity via an interaction with the T-cell receptor. (B) Selection criteria used to identify *P. gingivalis* peptides having the potential to engage with the peptide-binding groove of I-A^b^. Peptides that passed both of the inclusion criteria were synthesized and numbered 1 through 53.

The amino acid sequence, relative sequence position and W-R score assigned to each candidate peptide are provided in Table 1. Our stringent selection criteria gave us a total of 53 peptides divided between Kgp (14 peptides), RgpA (15 peptides), shared Kgp/RgpA (13 peptides), OMP40 (six peptides) and OMP41 (five peptides) that were subsequently synthesized and used to immunize C57BL/6 mice expressing the I-A^b^ molecule on APCs (Fig. 1B).

**Table 1:** Amino acid sequence and Wilson–Rudensky (W-R) scores associated with the selected 53 peptides

Peptide no.	Protein	AA at P1	−P2	−P1	P1	P2	P3	P4	P5	P6	P7	P8	P9	W-R score
1	Kgp	18	G	L	Y (30)	A	Q	S (12)	A	K (01)	I (02)	K	L (06)	51
2	Kgp	78	G	E	V (07)	G	S	P (30)	E	V (06)	P (00)	A	V (14)	57
3	Kgp	238	D	L	Y (30)	N	T	P (30)	V	R (00)	M (01)	L	V (14)	75
4	Kgp	295	K	K	Y (30)	N	D	G (05)	L	A (16)	A (09)	S	A (09)	69
5	Kgp	377	K	S	Y (30)	L	E	K (00)	A	L (02)	L (10)	I	A (09)	51
6	Kgp	390	D	S	Y (30)	W	N	P (30)	K	I (09)	G (05)	Q	Q (03)	77
7	Kgp	469	D	K	Y (30)	F	L	A (13)	I	G (07)	N (14)	C	C (01)	65
8	Kgp	639	Y	G	T (00)	G	V	A (13)	N	A (16)	S (07)	G	V (14)	50
9	Kgp	705	L	K	W (04)	D	A	P (30)	S	A (16)	K (00)	K	A (09)	59
10	Kgp/RgpA	925/901	V	K	Y (30)	T	A	G (05)	V	S (03)	P (00)	K	V (14)	52
11	Kgp/RgpA	988/968	E	S	F (16)	E	N	G (05)	I	P (21)	A (09)	S	W (00)	51
12	Kgp/RgpA	1011/991	K	P	G (00)	N	A	P (30)	G	I (09)	A (09)	G	Y (02)	50
13	Kgp/RgpA	1076/1056	A	V	Y (30)	A	S	S (12)	T	G (07)	N (14)	D	A (09)	72
14	Kgp/RgpA	1101/1081	K	G	V (07)	R	S	P (30)	E	A (16)	I (02)	R	G (03)	58
15	Kgp/RgpA	1286/1266	T	E	A (03)	N	G	A (13)	K	P (21)	Q (02)	S	V (14)	53
16	Kgp/RgpA	1300/1280	R	T	V (07)	D	L	P (30)	A	G (07)	T (04)	K	Y (02)	50
17	Kgp/RgpA	1300/1280	R	T	V (07)	D	L	P (30)	A	G (07)	T (04)	K	Y (02)	50
18	Kgp/RgpA	1314/1294	R	H	Y (30)	N	C	S (12)	D	L (02)	N (14)	Y	I (02)	60
19	Kgp/RgpA	1332/1312	T	M	G (00)	G	S	P (30)	T	P (21)	T (04)	D	Y (02)	57
20	Kgp/RgpA	1404/1384	N	L	K (01)	A	Q	P (30)	D	G (07)	G (05)	D	V (14)	57
21	Kgp	1497	N	F	L (04)	I	T	P (30)	K	V (06)	E (07)	G	A (09)	56
22	Kgp	1510	I	T	Y (30)	K	V	G (05)	S	P (21)	G (05)	L	P (00)	61
23	Kgp	1549	M	T	Y (30)	T	Q	G (05)	G	A (16)	N (14)	L	T (02)	67
24	Kgp	1574	R	H	Y (30)	N	C	T (03)	D	V (06)	L (10)	G	I (02)	51
25	Kgp/RgpA	1618/1598	T	T	Y (30)	R	D	A (13)	G	M (00)	S (07)	A	Q (03)	53
26	Kgp/RgpA	1636/1616	V	K	Y (30)	A	A	G (05)	V	S (03)	P (00)	K	V (14)	52
27	RgpA	68	P	T	Y (30)	T	E	G (05)	V	N (01)	L (10)	S	E (06)	52
28	RgpA	151	A	T	L (04)	D	D	P (30)	F	I (09)	L (10)	R	D (03)	56
29	RgpA	182	R	I	Y (30)	T	E	I (01)	T	V (06)	A (09)	V	S (06)	52
30	RgpA	220	M	N	Y (30)	E	P	G (05)	R	Y (00)	T (04)	P	V (14)	53
31	RgpA	320	Q	V	Y (30)	G	Q	I (01)	V	G (07)	N (14)	D	H (02)	54
32	RgpA	376	S	A	E (00)	G	G	P (30)	S	A (16)	D (01)	N	G (03)	50
33	RgpA	473	G	D	F (16)	L	F	S (12)	M	P (21)	C (00)	F	A (09)	58
34	RgpA	475	F	L	F (16)	S	M	P (30)	C	F (02)	A (09)	E	A (09)	66
35	RgpA	490	Q	K	D (01)	G	K	P (30)	T	G (07)	T (04)	V	A (09)	51
36	RgpA	563	T	V	F (16)	G	D	P (30)	S	L (02)	L (10)	V	R (03)	61
37	RgpA	599	C	D	Y (30)	N	G	A (13)	I	A (16)	T (04)	I	S (06)	69
38	RgpA	747	D	Q	Y (30)	G	Q	V (04)	I	P (21)	S (07)	D	T (02)	64
39	RgpA	774	F	E	Y (30)	T	V	P (30)	E	N (01)	A (09)	D	P (00)	70
40	RgpA	1100	K	T	V (07)	D	L	P (30)	A	G (07)	T (04)	K	Y (02)	50
41	RgpA	1396	L	K	W (04)	E	A	P (30)	S	A (16)	K (00)	K	T (02)	52
42	RgpA	1419	L	F	V (07)	T	I	E (00)	P	A (16)	N (14)	D	V (14)	51
43	OMP40	137	V	F	H (01)	I	I	P (30)	W	A (16)	G (05)	I	G (03)	55
44	OMP40	267	P	E	P (00)	T	Q	P (30)	T	V (06)	T (04)	R	V (14)	54
45	OMP40	299	N	V	Y (30)	N	T	A (13)	E	Y (00)	A (09)	K	T (02)	54
46	OMP40	262	S	C	P (00)	E	C	P (30)	E	P (21)	T (04)	Q	P (00)	55
47	OMP40	207	G	K	A (03)	D	F	P (30)	V	M (00)	A (09)	T	A (09)	51
48	OMP40	317	V	G	Y (30)	A	D	E (00)	K	T (06)	G (05)	T	A (09)	50
49	OMP41	83	G	K	Y (30)	H	S	P (30)	F	F (02)	A (09)	T	R (03)	74
50	OMP41	209	P	V	F (16)	E	D	P (30)	A	G (07)	R (01)	Y	Y (02)	56
51	OMP41	10	L	T	L (04)	V	G	A (13)	I	A (16)	L (10)	N	A (09)	52
52	OMP41	185	D	F	V (07)	I	E	A (13)	Q	A (16)	A (09)	H	S (06)	51
53	OMP41	149	V	G	Y (30)	Q	H	K (00)	F	I (09)	G (05)	S	E (06)	50

Single letter amino acid code is used. Peptide sequence and location within a given protein is based on *Porphyromonas gingivalis* strain ATCC 39377. Peptides found in both RgpA and Kgp are indicated as Kgp/RgpA. Numbers in parentheses are individual values associated with that particular amino acid occupying that particular position and predicted to engage pocket P1, P4, P6, P7 or P9 of the MHC class II I-A^b^ molecule. The W-R score is a summation of these values.

### *Porphyromonas gingivalis* re-stimulates effector/memory CD4^+^ T cells when primed with RgpA and Kgp but not with OMP40- or OMP41-derived peptides

**Figure 2:**
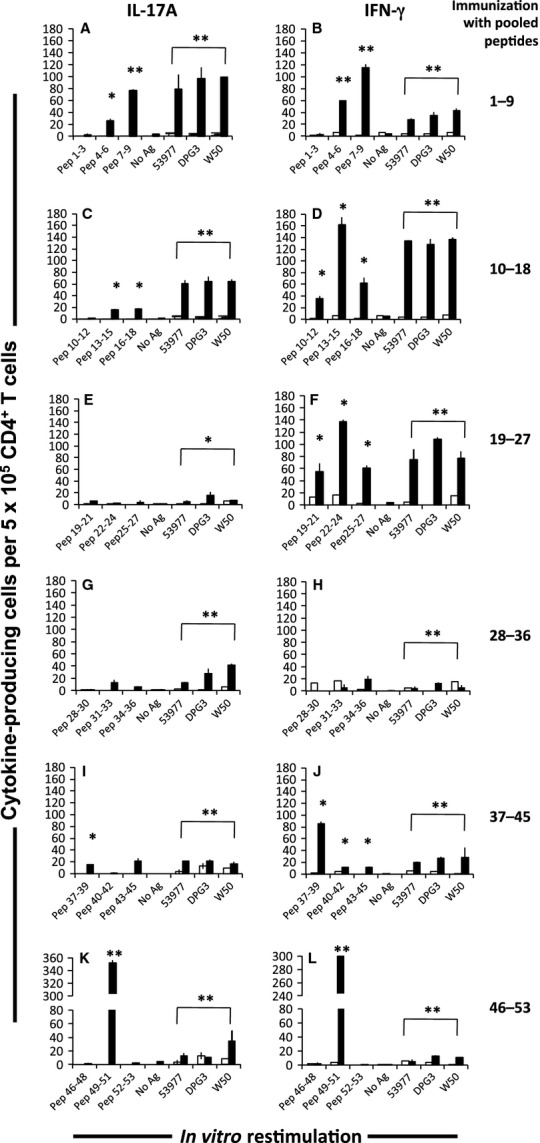
Secretion of interferon-γ (IFN-γ) or interleukin-17A (IL-17A) by antigen-specific CD4^+^ T cells following *in vitro* recall stimulation with pooled peptide fractions or *Porphyromonas gingivalis*. CD4^+^ T cells were purified from mice inoculated with pooled peptide in conjunction with lipopolysaccharide and incomplete Freund's adjuvant. 5 × 10^5^ CD4^+^ T cells were co-cultured with 3 × 10^5^ irradiated naive splenocytes in the presence of an appropriate pooled peptide fraction or *P. gingivalis* as recall antigen. Black bars represent the number of CD4^+^ T cells expressing IFN-γ or IL-17A per well. The recall antigen used is indicated below each bar and the makeup of the pooled peptide used to inoculate each mouse is given to the right of each graph pair. Mice inoculated with lipopolysaccharide plus incomplete Freund's adjuvant were the source of CD4^+^ T cells used as negative controls (open bars). Data represent the means of duplicate wells. One-tailed Student's *t*-test was used to determine if recall sample means were significantly larger than the re-stimulation without antigen (negative control: No Ag). *P* values < 0.05 or < 0.01 are indicated by a single or double asterisk, respectively. Data for all three *P. gingivalis* strains were pooled for this analysis.

Subcutaneous immunization with 3 × 10^9^ CFU live or killed *P. gingivalis* did not elicit effector/memory CD4^+^ T cells that could be re-stimulated with a test set of six peptides with W-R score > 60, including peptide 13 (see below). Therefore, C57BL/6 mice were subcutaneously immunized with sets of nine different pooled peptides plus *E. coli* lipopolysaccharide in incomplete Freund's adjuvant. The objective was to test whether the peptides identified with the W-R algorithm were able to stimulate the CD4^+^ T-cell repertoire of C57BL/6 mice, which could be re-stimulated *in vitro* with *P. gingivalis*. Purified CD4^+^ T cells were incubated with either irradiated naive splenocytes plus peptide sub-pools comprising three constituent peptides or *P. gingivalis*. We observed that *P. gingivalis* induced IFN-γ and IL-17A expression in effector/memory CD4^+^ T-cell clones primed with Kgp peptides 1 to 9 (Fig. 2A,B) or primed with RgpA/Kgp shared peptides 10 to 18 (Fig. 2C,D). Peptides 19 to 27, which are found in both RgpA and Kgp, primed effector/memory CD4^+^ T cells that when re-stimulated with *P. gingivalis* or peptide sub-pool comprising peptides 22 to 24 expressed primarily IFN-γ (Fig. 2F), but not IL-17A (Fig. 2E). Peptides 28 to 36 were not considered immunogenic because only a weak response was seen when CD4^+^ T cells from immunized mice were re-stimulated in the presence of *P. gingivalis* or sub-pool peptides (Fig. 2G,H).

Pooled peptides from OMP40 and OMP41 (peptides 46–53) were very efficient in priming a set of effector/memory CD4^+^ T cells specific for peptides 49–51. These cells expressed either IFN-γ or IL-17A. However, such subsets of effector/memory CD4^+^ T-cell specificities could not be re-stimulated by *P. gingivalis* (Fig. 2K,L). Further examination of peptides 49, 50 and 51 demonstrated that the epitope in peptide 51, despite being highly immunogenic, falls within the putative signal peptide of OMP41 (data not shown). It is highly likely that this epitope is removed during OMP41 maturation and may not be available for loading onto MHC class II molecules during processing of *P. gingivalis* by APCs. Therefore, notwithstanding the extremely efficient priming and re-activation of effector/memory CD4^+^ T cells, peptide 51 was not suitable for incorporating into a pMHCII tetramer. Similarly, one or more of peptides 37, 38 or 39 was found to be immunogenic when CD4^+^ T cells from immunized mice were re-stimulated with this sub-pool, but not when re-stimulated with *P. gingivalis* (Fig. 2I,J).

### *Porphyromonas gingivalis* re-stimulates CD4^+^ T cells when initially primed with peptide 7 (pKgp) and peptide 13 (pR/Kgp)

**Figure 3:**
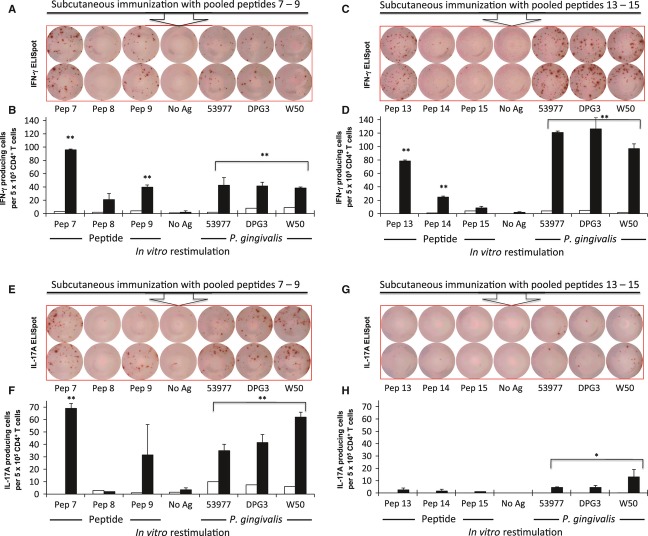
*In vitro* activation of antigen-specific CD4^+^ T cells indicates that peptide 7 and peptide 13 are immunogenic T-cell epitopes capable of engaging the peptide-binding groove of I-A^b^. Mice were inoculated with the indicated pooled peptide fractions with lipopolysaccharide plus incomplete Freund's adjuvant. CD4^+^ T cells were purified from these animals and recalled with individual peptides or *Porphyromonas gingivalis* and assayed for interferon-γ (IFN-γ) or interleukin-17A (IL-17A) expression by ELISpot as described in the legend to Fig. 2. Images of ELISpot wells are depicted in (A,C,E,G) with graphs representing the number of CD4^+^ T cells expressing IFN-γ or IL-17A shown in (B,D,F,H). Black bars represent data from inoculated mice; open bars represent data from negative control mice. Data represent the means of two wells. Student's *t*-test was used to test significance as described in the legend to Fig. 2.

We found three peptide sub-pools that induced the priming of CD4^+^ T cells, which could be re-stimulated by *P. gingivalis* in ELISpot assays. To reveal the identities of the single immunodominant CD4^+^ T-cell epitope responsible, we immunized C57BL/6 mice with the appropriate three-peptide sub-pools (7–9; 13–15; 22–24). When C57BL/6 mice were primed subcutaneously with pooled peptides 7–9, more effector/memory CD4^+^ T cells released IFN-γ and IL-17A when re-stimulated with individual peptide 7 (Kgp_467-477_; DKYFLAIGNCC) than with peptide 8 or 9 (Fig. 3A,C). This demonstrates that peptide 7 from Kgp is the dominant immunogenic component of the 7–9 peptide sub-pool. Interestingly, peptide 9 was also able to re-stimulate effector/memory CD4^+^ T cells to release IFN-γ and IL-17A, but the magnitude of the response was less than that seen with peptide 7. Peptides 13–15 primed effector/memory CD4^+^ T cells to release IFN-γ when re-stimulated with peptide 13 (RgpA_1054–1064_/Kgp_1074–1084_; AVYASSTGNDA) but not with peptides 14 or 15 (Fig. 3B). This determines that peptide 13 found in both Kgp and RgpA is the immunogenic component of the 13–15 peptide sub-pool. Moreover, *P. gingivalis* was able to re-stimulate peptide 7-specific and peptide 13-specific effector/memory CD4^+^ T cells to produce IFN-γ confirming that both these peptides are displayed on I-A^b^ molecules following *P. gingivalis* processing by APCs (Fig. 3A,B). However, only peptide 7 induced IL-17A-producing effector/memory CD4^+^ T cells (Fig. 3C). Subcutaneous immunization with peptides 13–15 did not result in effector/memory CD4^+^ T cells capable of producing IL-17A when re-stimulated with peptide 13 alone or with *P. gingivalis* (Fig. 3D). Peptides 22, 23 and 24 did not elicit effector/memory CD4^+^ T-cell responses above background when re-stimulated with individual peptide or *P. gingivalis* (data not shown). The response seen in Fig. 2F may be a result of the cumulative effect of individual weak responses by peptides 22, 23 and 24. RgpA/Kgp shared peptides 22, 23 and 24 were, therefore, deemed not to be suitable CD4^+^ T-cell epitopes for further study. In summary, these ELISpot results indicate that peptide 7 and peptide 13 are expressed by the *P. gingivalis* strains ATCC 53977, W50 or DPG3, are processed by APCs, and are loaded on I-A^b^ molecules to effectively re-stimulate peptide-specific effector/memory CD4^+^ T cells in C57BL/6 mice. Therefore, peptide 7 (pKgp) and 13 (pR/Kgp) are immunodominant CD4^+^ T-cell epitopes in this mouse strain.

### Oral colonization with *P. gingivalis* activates RgpA- and Kgp-specific CD4^+^ T cells

**Figure 4:**
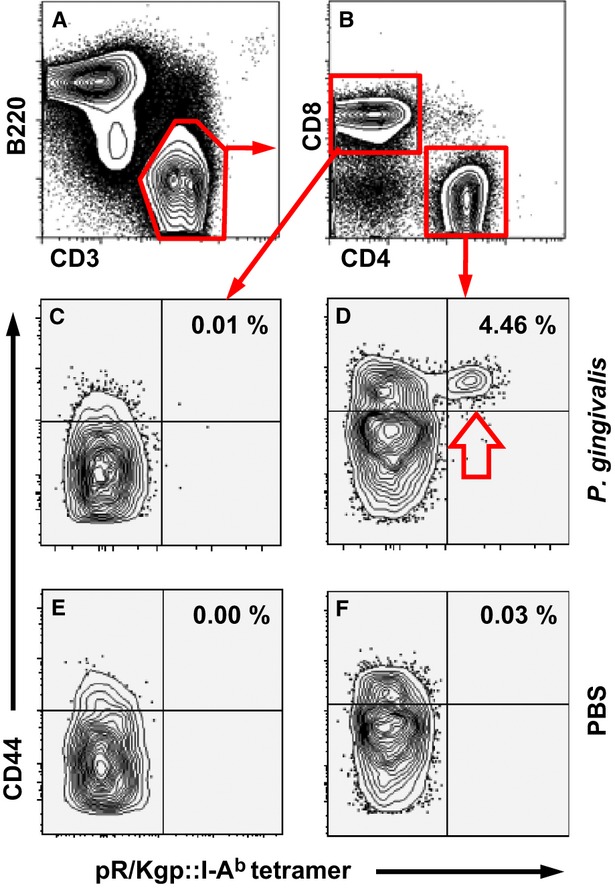
pR/Kgp::I-Ab tetramer identifies pR/Kgp-specific CD4^+^ T cells. Mice were orally inoculated and colonized with *Porphyromonas gingivalis* ATCC 53977 or sham-treated with phosphate-buffered saline (PBS) 21 days before harvesting draining cervical lymph nodes. pR/Kgp-specific CD4^+^ T cells were enriched using immunomagnetic-positive selection before cell surface staining to discriminate various lymphocyte subsets. (A) Representative FACS contour plots separating B cells (B220^+^) from T cells (CD3^+^). (B) CD3^+^ T cells are sorted into CD8^+^ (control, C,E) and CD4^+^ (test D,F) T cells. CD3^+^ CD4^+^ and CD3^+^ CD8^+^ T cells from *P. gingivalis* (C,D) and PBS (E,F) orally treated mice. The percentage of pR/Kgp-specific CD4^+^ T cells found in each sample is given. FACS plots depicted were typical of three independent experiments.

We designed and engineered two tetrameric I-A^b^ molecules (tetramers), one displaying pKgp and the other displaying pR/Kgp, and bound them to phycoerythrin-conjugated streptavidin to generate pKgp::I-A^b^ and pR/Kgp::I-A^b^ tetramers (data not shown). These tetramers were used to identify antigen-experienced CD4^+^ T cells specific for RgpA and/or Kgp in single-cell suspensions prepared from the draining cervical lymph nodes of C57BL/6 mice orally colonized with *P. gingivalis*. Oral colonization with *P. gingivalis* over 21 days, induced the proliferation and clonal expansion of RgpA- and Kgp-specific CD4^+^ T cells. Phycoerythrin-conjugated pR/Kgp::I-A^b^ tetramer discerned antigen-experienced (CD44^high^) RgpA- and Kgp-specific CD4^+^ T cells very effectively and very efficiently (Fig. 4D, open arrow). The frequency of CD3^+^ CD4^+^ CD44^high^ pR/Kgp::I-A^b +^ T cells in mice orally colonized with *P. gingivalis* was significantly higher than that found in sham-inoculated mice (Fig. 4F), indicating significant clonal expansion. Furthermore, the very low frequency of CD8^+^ T cells stained by pR/Kgp::I-A^b^ tetramer is indicative of its specificity for RgpA- and Kgp-specific CD4^+^ T cells (Fig. 4C,E). As expected, very few naive pR/Kgp-specific CD4^+^ T cells (CD44^low^) were detected in samples prepared from the cervical lymph nodes of orally colonized or PBS-treated C57BL/6 mice (Fig. 4D,F). In contrast, the specificity of the pKgp::I-A^b^ tetramer (i.e. Kgp_467–477_ peptide) was not sufficiently high to discriminate pKgp-specific CD4^+^ T cells from background staining of CD4^+^ T cells in general and of CD8^+^ T cells, and would not be a suitable reagent for future studies (data not shown).

## Discussion

We have taken a paired computational/ELISpot approach to reveal immunodominant CD4^+^ T-cell epitopes within important virulence factors of the periodontal pathogen *P. gingivalis*. Identification of immunodominant CD4^+^ T-cell epitopes is of particular interest in the development of vaccines that promote an effective cell-based immunity against particular pathogens. Moreover, the characterization of immunodominant CD4^+^ T-cell epitopes within known antigens can add valuable insight into the adaptive B-cell humoral response.

We based our approach on the W-R algorithm that predicts the likelihood of a given peptide being displayed by the I-A^b^ MHC class II molecule present in the C57BL/6 mouse (Zhu *et al*., 2003). We found that relatively few peptides were able to trigger a CD4^+^ T-cell response as determined by ELISpot assays. Of the 53 peptides we selected based on an aggregate W-R score of greater than 50, only peptides 7, 9, 13, 51 and one of peptides 37, 38 or 39 were able to re-stimulate effector/memory CD4^+^ T cells in an antigen-specific manner when re-challenged with the matching individual peptides. The C57BL/6 mouse displays a unique MHC class II molecule, I-A^b^. Therefore, the repertoire of peptides that are capable of being presented is potentially narrower and less diverse compared with BALB/c mice (I-A^d^, I-E^d^), for example. It remains to be tested as to whether or not any of the 53 peptides can be displayed by human APCs. However, humans have a larger capacity to display a more diverse pool of CD4^+^ T-cell epitopes because of multiple MHC class II isotypes that are present on an APC of a single individual.

Surprisingly, we did not find suitable CD4^+^ T-cell epitopes within the two outer membrane proteins that we examined even though members of the OmpA superfamily have been shown to be particularly immunogenic. OmpA proteins generate robust humoral responses in a number of infectious diseases (Weiser & Gotschlich, 1991; Das *et al*., 1998; Hara *et al*., 2009). Ross *et al*. (2004) found that two soluble C-terminal fragments from OMP40 (213–380 aa) and OMP41 (224–391 aa) provided good protection against *P. gingivalis* infection when used as vaccines in a murine subcutaneous lesion model. Although none of our selected peptides fell within the OMP40 fragment tested, OMP41-derived peptides 44, 45, 46 and 48 all fall within the antigenic region identified by Ross *et al*. (2004). Failure to detect these epitopes may be indicative of differences in MHC class II presentation of peptides between the BALB/c mice used by Ross *et al*. (2004) in their protection study and the C57BL/6 mice used here. Our inability to find such epitopes within OMP40 and OMP41 may be due to the characteristics of the I-A^b^ groove compared with I-A^d^/I-E^d^. I-A^b^ was probably unable to display the peptides predicted by the W-R algorithm (Dongre *et al*., 2001; Zhu *et al*., 2003). Alternatively, with the exception of peptide 51, the T-cell receptor specificities for OMP40 and OMP41 peptides were absent from the T-cell receptor repertoire of C57BL/6 mice. We may have also disregarded the other OMP40 and OMP41 CD4^+^ T-cell epitopes because of the stringent selection criteria that we put in place to avoid peptides having viable alternative I-A^b^ binding registries. Indeed, we elected not to synthesize several peptides from the antigenic regions of OMP40 (one peptide) and OMP41 (four peptides), which had W-R scores greater than 50, but also had strong alternative I-A^b^ binding registries.

Interestingly, we did see a vigorous CD4^+^ T-cell response when mice were immunized with OMP41 peptides 49–51 and re-stimulated with peptide 51 (W-R score of 52), but this was not replicated when effector/memory CD4^+^ T cells were challenged with *P. gingivalis*. Further examination of peptide 51 (OMP41_8–18_; LTLVGAIALNA) indicated that it was located wholly within the predicted signal peptide sequence of OMP41 (1–21 aa). The peptide 51 sequence may, therefore, be cleaved and subsequently degraded during OMP41 maturation within *P. gingivalis*, and so would not be available for display by MHC class II molecules following phagocytosis and processing of *P. gingivalis* antigens by APCs.

Overall, relatively few immunodominant CD4^+^ T-cell epitopes would seem to reside within any given protein. Peptides eluted from MHC class II and identified with tandem mass spectrometric methods were representative of a diverse range of endogenous proteins in naive C57BL/6 mice (Dongre *et al*., 2001; Bozzacco *et al*., 2011). Most proteins only had one or two core peptides associated with the relevant MHC class II molecule. It may not be surprising therefore, that out of the 27 Kgp-derived peptides we selected, we only found two (Kgp_467–477_; DKYFLAIGNCC and Kgp_1074–1084_; AVYASSTGNDA) that were able to act as immunodominant CD4^+^ T-cell epitopes in ELISpot analyses. In their study, out of 8189 possible theoretical peptides of between 7 and 27 amino acids in length derived from the core antigenic region of HIV gag p24 (133–363 aa), Bozzacco *et al*. (2012) were only able to discern two actual peptides displayed on splenic dendritic cells isolated from F_1_ hybrid mice (I-A^b^, I-A^d^, I-E^d^). Both of these CD4^+^ T-cell epitopes were highly similar, and both probably share the same core MHC class II binding region.

We found that peptide 7 (Kgp_467–477_; DKYFLAIGNCC) and peptide 13 (RgpA_1054–1064_/Kgp_1074–1084_; AVYASSTGNDA) were both capable of acting as immunodominant CD4^+^ T-cell epitopes and, importantly, were available to naive CD4^+^ T cells following APC processing and display of *P. gingivalis* epitopes on I-A^b^ molecules. In terms of W-R score, peptide 7 (W-R score 65) ranked 10th out of the 53 peptides tested and peptide 13 (W-R score 72) ranked 4th overall. Both peptides scored well above our cutoff of 50. A high W-R score, however, does not necessarily translate into a peptide being an immunodominant CD4^+^ T-cell epitope. The W-R algorithm is based largely on displayed endogenous peptides and not those from pathogens. The mass spectrometry method used by Dongre *et al*. (2001) to identify these peptides may favour those of high relative abundance at the expense of those of lower abundance (Dongre *et al*., 2001; Zhu *et al*., 2003). For example, the W-R algorithm assigns a high probability score of occupying the deep pocket at P1 to the relatively bulky hydrophobic amino acids phenylalanine (score 16) and tyrosine (score 30). Of our 53 selected peptides, 30 had either a tyrosine or a phenylalanine residue occupying P1, including both peptide 7 and 13, yet peptide 51 with an unfavoured leucine (score 4) at this position was very efficient in priming a set of effector/memory CD4^+^ T cells. Interestingly, peptide 51 had probably a larger population of responding effector/memory CD4^+^ T cells compared with either peptide 7 or 13, despite having a lower W-R score. Although stability of a peptide in the binding groove of an MHC class II molecule is important, the magnitude of the CD4^+^ T-cell response is also related to the frequency of the naive CD4^+^ T-cell population capable of recognizing a given epitope (Moon *et al*., 2007; Moon & Jenkins, 2012). In this regard, the polyclonal population of peptide 7- or 13-specific naive CD4^+^ T cells that exists in C57BL/6 mice is probably smaller than that of peptide 51.

Peptide 7 (Kgp_467–477_; DKYFLAIGNCC) is located within the putative protease catalytic domain of Kgp and during *P. gingivalis* infection may act as an immunodominant CD4^+^ T-cell epitope providing T helper type 2 help to a B-cell-mediated humoral response to Kgp-derived antigen (Kuboniwa *et al*., 2001). When used as a DNA vaccine in a lethal intraperitoneal mouse model, the catalytic domain of Kgp induced a response that prolonged survival rates by 43% following injection of live *P. gingivalis* (Kuboniwa *et al*., 2001). The location of peptide 13 (RgpA_1054–1064_/Kgp_1074–1084_; AVYASSTGNDA) within the adhesin domain A1 of both Kgp and RgpA may also be immunologically relevant. Vaccination with the RgpA-Kgp proteinase–adhesin complex provided protection against *P. gingivalis*-induced alveolar bone loss in a BALB/c periodontal mouse model (O'Brien-Simpson *et al*., 2005), and in a rat periodontitis model (Rajapakse *et al*., 2002). However, further delineation of the RgpA-Kgp proteinase–adhesin complex found that a recombinant adhesin domain A1 fragment that excluded peptide 13 still conferred protection against *P. gingivalis* infection in a subcutaneous lesion BALB/c mouse model (Frazer *et al*., 2006). Although yet to be tested in C57BL/6 mice, these data suggest that peptide 13 does not play a role in providing this protection in BALB/c mice. Qualitative differences in epitope presentation by different murine MHC class II molecules are likely.

In an effort to characterize T-cell epitopes within the RgpA-Kgp proteinase–adhesin complex, Tam *et al*. (2008) found a number of 15-mer peptides that could trigger T-cell responses in BALB/c mice, although the assay used did not differentiate between CD4^+^ or CD8^+^ T-cell epitopes. Nonetheless, 15-mers encompassing peptide 7 or 13 were not immunogenic in their assays and the immunodominant T-cell epitope identified by Tam *et al*. (2008) did not score above the threshold we set in our W-R analysis. We did test two peptides (4 and 5) that lay within immunogenic 15-mers identified by Tam *et al*. (2008), but neither were immunogenic in our C57BL/6 mouse-based assay. It should be reiterated that these differences may be related to how our peptide 4 and 5 are presented either by I-A^d^/I-E^d^ in BALB/c or I-A^b^ in C57BL/6 mice.

We selected, engineered and produced a pMHCII tetramer complex based on the sequence of peptide 13 (RgpA_1054–1064_/Kgp_1074–1084_; AVYASSTGNDA). Importantly, our data showed that we could use this tetramer to detect expanded effector/memory pR/Kgp (peptide 13) -specific CD4^+^ T cells with high specificity in draining cervical lymph nodes following *P. gingivalis* infection of the oral mucosa of C57BL/6 mice. This result is significant because it will allow us to identify, *in vivo* and at the single-cell level, immunodominant CD4^+^ T helper cell subsets in mice carrying the I-A^b^ molecule. Previous studies characterizing the contribution of T helper cells to periodontitis in mice have been associative in nature. For example, CD4^+^ T-cell phenotypes were assigned by examining cytokines secreted by total T-cell populations isolated from *P. gingivalis-*infected mice using ELISA (Orth *et al*., 2011), or responses to *P. gingivalis* antigens following ELISpot experiments (reviewed in Pathirana *et al*., 2010).

The pR/Kgp (peptide 13; AVYASSTGNDA) sequence is conserved in all *P. gingivalis* strains for which *RgpA* and *Kgp* sequence information is available, including the fimbriated (ATCC 53977, ATCC 33277, W50, W83, 381 and TDC60) and afimbriated (DPG3) strains that are capable of eliciting alveolar bone destruction in murine periodontitis models (Baker *et al*., 2000a,b2000b). The pR/Kgp:I-Ab tetramer we engineered will have broad utility because of unparalleled specificity in detecting CD4^+^ T cells after priming induced by mucosal colonization with several important *P. gingivalis* strains. With this tool we can unambiguously determine the kinetics of activation and phenotype of CD4^+^ T cells and conclusively define the I-A^b^-restricted T helper cell response against two important virulence factors of *P. gingivalis*.

## References

[b1] Baker PJ, Dixon M, Evans RT, Dufour L, Johnson E, Roopenian DC (1999). CD4^+^ T cells and the proinflammatory cytokines gamma interferon and interleukin-6 contribute to alveolar bone loss in mice. Infect Immun.

[b2] Baker PJ, Dixon M, Roopenian DC (2000a). Genetic control of susceptibility to *Porphyromonas gingivalis*-induced alveolar bone loss in mice. Infect Immun.

[b3] Baker PJ, Dixon M, Evans RT, Roopenian DC (2000b). Heterogeneity of *Porphyromonas gingivalis* strains in the induction of alveolar bone loss in mice. Oral Microbiol Immunol.

[b4] Baker PJ, Howe L, Garneau J, Roopenian DC (2002). T cell knockout mice have diminished alveolar bone loss after oral infection with *Porphyromonas gingivalis*. FEMS Immunol Med Microbiol.

[b5] Bozzacco L, Yu H, Zebroski HA (2011). Mass spectrometry analysis and quantitation of peptides presented on the MHC II molecules of mouse spleen dendritic cells. J Proteome Res.

[b6] Bozzacco L, Yu H, Dengjel J (2012). Strategy for identifying dendritic cell-processed CD4^+^ T cell epitopes from the HIV gag p24 protein. PLoS ONE.

[b7] Byrne SJ, Dashper SG, Darby IB, Adams GG, Hoffmann B, Reynolds EC (2009). Progression of chronic periodontitis can be predicted by the levels of *Porphyromonas gingivalis* and *Treponema denticola* in subgingival plaque. Oral Microbiol Immunol.

[b8] Das M, Chopra AK, Cantu JM, Peterson JW (1998). Antisera to selected outer membrane proteins of *Vibrio cholerae* protect against challenge with homologous and heterologous strains of *V. cholerae*. FEMS Immunol Med Microbiol.

[b9] Dixon DR, Bainbridge BW, Darveau RP (2004). Modulation of the innate immune response within the periodontium. Periodontol 2000.

[b10] Dongre AR, Kovats S, deRoos R (2001). *In vivo* MHC class II presentation of cytosolic proteins revealed by rapid automated tandem mass spectrometry and functional analyses. Eur J Immunol.

[b11] Ebersole JL, Taubman MA, Smith DJ, Frey DE, Haffajee AD, Socransky SS (1987). Human serum antibody responses to oral microorganisms. IV. Correlation with homologous infection. Oral Microbiol Immunol.

[b12] Frazer LT, O'Brien-Simpson NM, Slakeski N (2006). Vaccination with recombinant adhesins from the RgpA–Kgp proteinase–adhesin complex protects against *Porphyromonas gingivalis* infection. Vaccine.

[b13] Gemmell E, Yamazaki K, Seymour GJ (2007). The role of T cells in periodontal disease: homeostasis and autoimmunity. Periodontol 2000.

[b14] Hara Y, Mohamed R, Nathan S (2009). Immunogenic *Burkholderia pseudomallei* outer membrane proteins as potential candidate vaccine targets. PLoS ONE.

[b15] Kuboniwa M, Amano A, Shizukushi S, Nakagawa I, Hamada S (2001). Specific antibodies to *Porphyromonas gingivalis* lys-gingipain by DNA vaccination inhibit bacterial binding to hemoglobin and protect mice from infection. Infect Immun.

[b16] Lamont RJ, Jenkinson HF (2000). Subgingival colonization by *Porphyromonas gingivalis*. Oral Microbiol Immunol.

[b17] Miyachi K, Ishihara K, Kimizuka R, Okuda K (2007). Arg-gingipain A DNA vaccine prevents alveolar bone loss in mice. J Dent Res.

[b18] Moon JJ, Jenkins MK (2012). The role of naive T cell precursor frequency and recruitment in dictating immune response magnitude. J Immunol.

[b19] Moon JJ, Chu HH, Pepper M (2007). Naive CD4^+^ T Cell frequency varies for different epitopes and predicts repertoire diversity and response magnitude. Immunity.

[b20] Moon JJ, Chu HH, Hataye J (2009). Tracking epitope-specific cells. Nat Protoc.

[b21] Naito M, Hirakawa H, Yamashita A (2008). Determination of the genome sequence of *Porphyromonas gingivalis* strain ATCC 33277 and genomic comparison with strain W83 revealed extensive genome rearrangements in *P. gingivalis*. DNA Res.

[b22] O'Brien-Simpson N, Veith PD, Dashper SG, Reynolds EC (2003). *Porphyromonas gingivalis* gingipains: the molecular teeth of a microbial vampire. Curr Protein Pept Sci.

[b23] O'Brien-Simpson NM, Pathirana RD, Paolini RA (2005). An immune response directed to proteinase and adhesin functional epitopes protects against *Porphyromonas gingivalis*-induced periodontal bone loss. J Immunol.

[b24] Oliver RC, Brown LJ (1993). Periodontal diseases and tooth loss. Periodontol 2000.

[b25] Oliver RC, Brown LJ, Loe H (1998). Periodontal diseases in the United States population. J Periodontol.

[b26] Orth RK, O'Brien-Simpson NM, Dashper SG, Reynolds EC (2011). Synergistic virulence of *Porphyromonas gingivalis* and *Treponema denticola* in a murine periodontitis model. Mol Oral Microbiol.

[b27] Pathirana RD, O'Brien-Simpson NM, Reynolds EC (2010). Host immune responses to *Porphyromonas gingivalis* antigens. Periodontol 2000.

[b28] Rajapakse PS, O'Brien-Simpson NM, Slakeski N, Hoffmann B, Reynolds EC (2002). Immunization with the RgpA-Kgp proteinase-adhesin complexes of *Porphyromonas gingivalis* protects against periodontal bone loss in the rat periodontitis model. Infect Immun.

[b29] Ross BC, Czajkowski L, Vandenberg KL (2004). Characterization of two outer membrane protein antigens of *Porphyromonas gingivalis* that are protective in a murine lesion model. Oral Microbiol Immunol.

[b30] Sharma DC, Prasad SB, Karthikeyan BV (2007). Vaccination against periodontitis: the saga continues. Expert Rev Vaccines.

[b31] Socransky SS, Haffajee AD, Cugini MA, Smith C, Kent RL (1998). Microbial complexes in subgingival plaque. J Clin Periodontol.

[b32] Tam V, O'Brien-Simpson NM, Pathirana RD, Frazer LT, Reynolds EC (2008). Characterization of T cell responses to the RgpA-Kgp proteinase–adhesin complexes of *Porphyromonas gingivalis* in BALB/c mice. J Immunol.

[b33] Tran SD, Rudney JD (1999). Improved multiplex PCR using conserved and species-specific 16S rRNA gene primers for simultaneous detection of *Actinobacillus actinomycetemcomitans*
*Bacteroides forsythus* and *Porphyromonas gingivalis*. J Clin Microbiol.

[b34] Van Dyke TE, Offenbacher S, Place D, Dowell VR, Jones J (1988). Refractory periodontitis: mixed infection with *Bacteroides gingivalis* and other unusual *Bacteroides* species. A case report. J Periodontol.

[b35] Weiser JN, Gotschlich EC (1991). Outer membrane protein A (OmpA) contributes to serum resistance and pathogenicity of *Escherichia coli* K-1. Infect Immun.

[b36] Yang HW, Huang YF, Chou MY (2004). Occurrence of *Porphyromonas gingivalis* and *Tannerella forsythensis* in periodontally diseased and healthy subjects. J Periodontol.

[b37] Zhu Y, Rudensky AY, Corper AL, Teyton L, Wilson IA (2003). Crystal structure of MHC Class II I-A^b^ in complex with a human CLIP peptide: prediction of an I-A^b^ peptide-binding motif. J Mol Biol.

